# The effect of masks on the emotion perception of a facial crowd

**DOI:** 10.1038/s41598-023-41366-0

**Published:** 2023-08-31

**Authors:** Jieun Cho, Hee Yeon Im, Young Jun Yoon, Sung Jun Joo, Sang Chul Chong

**Affiliations:** 1 Graduate Program in Cognitive Science, Yonsei University, Seoul, South Korea; 2 Department of Psychology, University of British Columbia, Vancouver, Canada; 3 Department of Psychology, Pusan National University, Busan, South Korea; 4 Department of Psychology, Yonsei University, Seoul, South Korea

**Keywords:** Psychology, Human behaviour

## Abstract

The present study investigated the effect of facial masks on people’s ability to perceive emotions in crowds. We presented faces with the bottom halves occluded by masks or full faces without occlusion. In two sequentially presented crowds, we varied the number of faces, emotional valence, and intensity of facial expressions, examining the impact of masks on the perception of crowd emotion. Participants reported which of the two crowds they would avoid based on the crowds’ average emotions. The participants’ ability to judge the average emotion of a crowd, especially a crowd expressing happiness, was impaired when the crowd wore masks. For faces covered by masks, crowd emotion judgments were more negatively biased than those without masks. However, participants could still distinguish the emotional intensities of a crowd wearing masks above chance. Additionally, participants responded more quickly to a crowd with more people without compromising accuracy, despite the perceptual challenges imposed by facial masks. Our results suggest that under ambiguous social situations in which individuals’ emotions are partially hidden by masks, a large group may provide stronger social cues than a small group, thereby promoting communication and regulating social behaviors.

## Introduction

Due to the COVID-19 pandemic, mask-wearing has become a requirement worldwide. As a result, many social interactions have been allowed only when people wear masks. However, wearing masks conceals the visual information conveyed by the lower half of a face, especially the region containing the mouth and nose. Like COVID-19 itself, mask-wearing will likely not be a permanent reality; however, it may not be eliminated since people have learned this new habit as a potential response to future outbreaks (https://www.nature.com/articles/d41586-021-01394-0). Although the impacts of mask-wearing on public health are under extensive investigation as an effective nonpharmacologic intervention to reduce the spread of infection, the potential impacts of continued mask-wearing on social cognition and behaviors related to everyday activities have not been evaluated.

Recent studies have reported that perceiving faces obstructed by masks hampered adults’ recognition of facial identity^1^ and emotion^2,3^. Similar outcomes were found in school-aged children^4^. Specifically, Freud et al.^1^ suggested that perceiving faces with masks that cover the lower half of a face engaged a local, feature-based processing and significantly reduced holistic processing, which is considered a hallmark of face perception^5–7^. Regarding emotion perception, both adults and children showed reduced accuracy, although they could still categorize the emotion expressed by a masked face better than chance^2–4^. In these previous studies, observers identified the emotion of a face wearing a mask among discrete emotional labels (e.g., happy, angry, neutral) by engaging in categorical emotion perception^8^.

Labeling an emotion with a specific category modulates how people perceive facial expressions^9–12^. For example, two faces expressing emotions from distinct categories (e.g., happiness and sadness) tended to be distinguished more easily than two faces expressing emotions within the same category, even when the emotional intensity and physical differences were matched for both pairs of faces^9,10,12,13^. When some deterministic facial features of a face wearing a mask become ambiguous or unavailable, the perceived emotion of the face can be strengthened by verbal labels due to the categorical perception. For example, a masked face can be perceived as happier more easily once labeled as such. However, in many day-to-day interactions, labeling a facial emotion using a discrete category (e.g., “This person looks happy, and this person looks angry”) is not always practical given the high-dimensional taxonomy^14^ and continuous nature (mixed emotions^15^) of emotions and emotional experiences.

People in any social setting tend to share a consistent emotional state rather than expressing categorically different emotions, although the intensity and magnitude of the emotion may vary (e.g., joy at a rock concert, sadness at a funeral, fear of terrorism, or anger at a political or social demonstration). The overall mood of a crowd creates a meaningful context, providing a social-emotional signal that rapidly informs observers of appropriate behaviors in a social context^16–19^. As a member of a protest march or political demonstration, one might perceive anger as a prevalent emotion in the group. Among facial groups expressing various spectrums of anger, one might want to make a relative emotional judgment to ensure that they stay away from a group that looks infuriated to avoid any unpredictable violence. Therefore, extracting a crowd emotion is a useful social skill for comprehending and reacting appropriately to the social context of one’s surroundings^20^. Previous research has investigated whether this ability may stem from our ensemble coding ability. For example, averaging different faces’ emotions can be performed rapidly and efficiently without perceiving each face serially^21–23^. Due to noise cancellation, averaging facial expressions is also more robust against the noise produced by face inversion than processing a facial expression^24^.

When people see others’ faces covered by masks from a physical distance in public spaces, they often misinterpret the partially occluded facial expressions. Recent works on the perception of a mask-wearing face mainly examined close one-to-one interpersonal interactions and may not apply to the perception of a facial crowd. Therefore, it is still unknown how the new normal social stimuli (i.e., multiple upper halves of faces) influence the perceptual ability to extract crowd emotion and interpret the overall social ambiance. The present study investigated how covering the lower halves of faces influences people’s perceptual judgments of the emotions of crowds. We compared participants’ accuracy and response time (RT) according to three facial mask-wearing group conditions: when both crowds wore masks (*both* condition), when only one crowd wore masks (*either* condition), and when neither crowd wore masks (*neither* condition). We varied the number of faces to examine the effect of crowd size on participants’ judgment of a crowd’s emotion. More faces in crowds may increase challenges when the mask-wearing faces provide less emotion-related information. However, if partial information from the upper halves of faces can be sufficiently processed simultaneously, crowd size will not affect emotion perception in mask-wearing crowds. Instead, ensemble perception may even enhance emotion perception via noise canceling by averaging more samples, leading to improved perception of the overall emotion of the crowd.

In addition, we investigated how people perceived the overall emotion of a facial crowd wearing masks when the verbal labeling of emotion did not play a sufficient role. We did this by asking participants to choose which of two sequentially presented sets of faces they would like to avoid instead of asking participants to verbally label the emotion. Recent studies of a single face wearing a mask have shown that mask-wearing interfered with recognizing a happy face more strongly than an angry or fearful face^25^. Therefore, we also manipulated the emotional intensity and valence of the faces to examine whether mask-wearing influences the perception of positive crowd emotions more significantly than negative emotions.

## Methods

### Participants

A total of 40 people participated in this experiment: 20 undergraduate and graduate students (aged 21–32; 10 women and 10 men) from Yonsei University (Seoul, South Korea) and 20 undergraduate students (aged 18–27; 13 women and seven men) from Pusan National University (Busan, South Korea). We recruited more than twice as many participants as in previous studies using similar stimuli and tasks^22,24^. Furthermore, we analyzed our data using Bayesian methods, which are less influenced by the number of participants^26^. All participants had a normal color vision and normal or corrected-to-normal visual acuity. The experimental protocol was approved by the Institutional Review Boards at Yonsei and Pusan National Universities, and written informed consent was obtained according to their procedures. All methods were performed in accordance with the relevant guidelines and regulations by the Institutional Review Boards.

### Apparatus and stimuli

All stimuli were generated using the Psychophysics Toolbox Version 3 and its extensions (Brainard, 1997^27^; Pelli, 1997^28^) for MATLAB (MathWorks Inc., Natick, MA, USA). At Yonsei University, stimuli were displayed on a CRT monitor at a resolution of 1600 × 1200 with a refresh rate of 85 Hz. At Pusan National University, the same stimuli were displayed on a 27-in. full HD widescreen color monitor at a resolution of 1920 × 1080 with a refresh rate of 120 Hz. At both locations, participants were seated approximately 60 cm from the monitor with their heads on a head-chin rest inside a darkened room.

In each trial, we presented a set of faces, each of which expressed a specific emotion. Faces were morphed via FantaMorph software (Abrosoft), using face stimuli from the Yonsei Face Database^29^. The database consists of 344 photographs of 17 amateur Korean actors (nine women and eight men) displaying six basic emotions (anger, disgust, fear, happiness, sadness, and surprise) and a neutral facial expression.

The emotional intensity of each photograph in the original study^29^ was evaluated by a minimum of 177 Yonsei University students (177–212) for verification purposes. The evaluators categorized the emotion of each photograph with one of the seven facial labels and assessed its emotional intensity on a linear scale from 0 (very weak) to 10 (very strong). In the current study, we selected eight actors (three women and five men) and used each actor’s happy and angry faces for morphing. We only used the faces of individuals whose mouths were closed for the following reasons. Open-mouthed faces are generally perceived as more intense and extreme, resulting in higher valence and arousal levels^30^. They also exhibit a greater attentional advantage, especially when expressing anger and happiness^31–34^. Using faces with closed mouths only also allowed us to rule out the possibility that any difference between crowds with masked and unmasked people is explained solely by the difference in the perceptual saliency of the mouth area of the face.

Our selection criteria for the actors were as follows: (1) the face stimuli had similar intensity ratings between happy and angry; (2) the most frequent emotion judged by the observers (> 70%) was consistent with the emotional category the actor in the image intended to express (e.g., the most frequent “happy” response to a “happy” expression); (3) none of the other emotional labels were rated as high as happy (e.g., to prevent confusion with surprise or disgust) or angry (e.g., to prevent confusion with fear or sadness). Supplementary Table 1 details the observers’ emotional judgments for the eight face stimuli with both happy and angry emotions, including the emotion judgment frequency and rated emotional intensity (mean and standard deviation).

**Figure 1:**
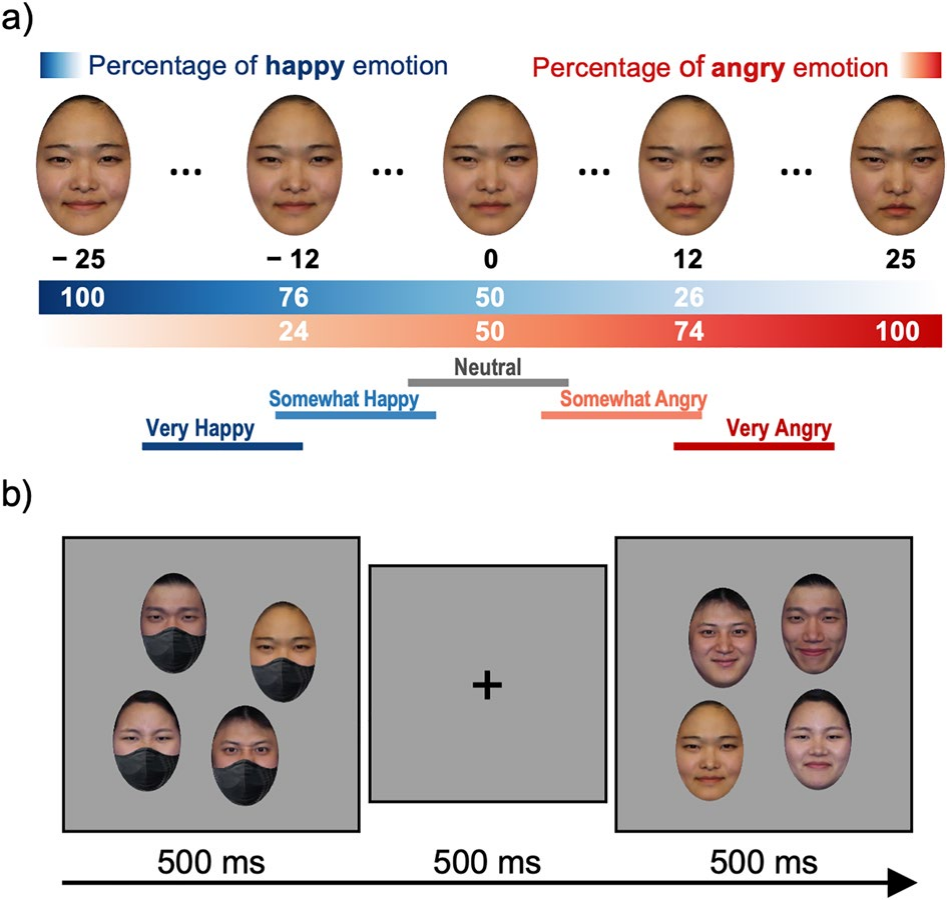
Stimuli and Procedure. *Note* (**a**) Emotional units produced by morphing happy and angry emotions (black numbers) and the morphing proportion of those emotions (white numbers in the blue and red spectrum bars). The horizontal color bars with labels ranging from Very Happy to Very Angry indicate the different emotional valence and intensity conditions used. (**b**) An example trial sequence. This example illustrates a trial of the *either* condition when the probe set represents happy faces with a set size of 4. Informed consent was obtained from amateur models for publishing the images.

By morphing the happy and angry face images with the same identity, we created 51 facial emotion morph levels ranging from − 25 (the happiest) to + 25 (the angriest). The morphed face images were linearly interpolated (in 2% increments) between the original happy and angry emotions. The same process was applied to the eight selected identities. The emotion level of 0 corresponded to a neutral face, which was a morph comprising 50% happy and 50% angry faces (Fig. 1a). Different faces were separated by emotional intensity units such that face 1 was one emotional unit happier than face 2. Therefore, the larger the separation between any two morphed faces in emotional units, the easier it was to discriminate them based on their emotional intensity.

Individual face images (2.27° × 3.37° visual angle in size at Yonsei University and 2.82° × 4.19° visual angle in size at Pusan National University) of a crowd were randomly positioned in an invisible frame (10° × 10° visual angle at the center of a gray background at Yonsei University and 12.4° × 12.4° visual angle at Pusan National University).

### Design and procedure

Our experiment had a 3 set sizes (one, four, and eight) × 4 emotions (very happy, somewhat happy, somewhat angry, and very angry) × 3 mask conditions of face sets (neither, either, and both) within-subject design, with 28 repetitions per condition. Therefore, there were 1008 trials in total, and the sequence of the trials was randomized for each participant. Participants took a short break after every 200 trials.

In each trial, two sets of faces were presented sequentially for 500 ms each (see Fig. 1b for a sample trial). Each set contained one, four, or eight different identity faces according to the set size condition. A black fixation cross on a blank screen was presented for 500 ms between the two displays of facial stimuli. One of the two displays always contained a neutral-emotion group with an average value of 0 emotional units as a control set. The probe set either had a mean of − 16 (very happy), − 8 (somewhat happy), + 8 (somewhat angry), or + 16 (very angry) emotion units. In other words, participants consistently compared a group of faces exhibiting one of the four average emotion intensities with a group of faces displaying a neutral emotion (where the average value was 0). Whether the control or probe set was presented first was randomly determined in every trial.

The minimum and maximum emotion intensities within the same emotion condition were the same across all set sizes, spanning a 10-unit range, except for the set size 1 condition where only the average value was chosen. Therefore, the happy conditions (including both somewhat happy and very happy conditions) contained only happy individual faces with varying intensities, while the angry conditions contained only angry individual faces. We also ensured that individual faces had emotion intensities that were distinct, uniformly distributed within the 10-unit range, and symmetrical around the average emotion. For example, if the emotion units used for the very angry condition in set size 4 were^11,15,17,21^, the corresponding emotion units for the same condition in set size 8 would be^11,12,14,15,17,18,20,21^, with varying middle values across trials. These manipulations were implemented to minimize the possibility that one or two faces with intense emotions would evoke faster responses due to saliency. For the neutral-emotion stimuli, the extreme values in the range were identical across trials: − 5 (happy) and 5 (angry). While maintaining the mean value of 0, the middle units varied across trials.

Importantly, there were three conditions, depending on whether the faces in a set (the probe or control set) wore masks. In the *both* condition, the faces wore masks in both the probe and control sets; in the *either* condition, only one set contained faces wearing masks, while the other set contained faces without masks; and in the *neither* condition, none of the sets contained faces wearing masks. Figure 1b presents a sample trial with the set size of four, when the control set (neutral; 0) was compared with the very happy crowd (− 16) and when only the faces in the control set were wearing masks (i.e., the *either* condition). In this figure, the control set containing individual faces with emotional units of [− 5, − 4, 4, 5] is presented first, followed by a blank screen and the probe set (average = −16) containing individual faces with emotional units of [− 21, − 19, − 13, − 11].

Participants were instructed to fixate on the center of the screen to view the two successive sets of multiple faces and make a keypress as accurately and quickly as possible to indicate which of two facial crowds they would rather avoid. We explicitly informed participants that the correct answer was the facial crowd showing a more negative emotion on average. Feedback was provided only during 20 practice trials and then removed for the main experiment. Participants’ responses to the first 20 practice trials were not included in the data analysis.

### Statistical analysis

We used the brms package^35^ for the Bayesian mixed model analysis and bayestestR packages^36^ to compare models. We fit models to predict the proportion of which facial crowd was judged as negative and the RTs, separately, depending on the set size, emotion, and mask conditions. The set size and the emotion predictors were treated as continuous predictors, while the facial mask predictor was treated as a categorical predictor. The response was binary—that is, 1 indicated a “more negative” response, and 2 indicated a “less negative” response for a probe set (a more expressive crowd) compared to a control set (a neutral crowd on average). Therefore, logistic regression models were fit to predict the proportion of “more negative” responses using the Bernoulli distribution with a logit link function. The RTs were fit to the shifted lognormal distribution with an identity link function as recommended in the literature^37^.

For each dependent variable, the full model consisted of the three predictors and a participant-level random intercept. The null model contained only the random intercept of participants. The null model was compared with other models comprising all possible combinations of the main effects or interaction terms. The model comparison results were reported as Bayes factors (BF_10_), which indicate the extent to which the data are better explained by one model than another^38^. For example, a BF_10_ of 3 indicates that the observed data are three times more probable under the alternative model, *H*_1_, than the null model, *H*_0_. As established in the previous literature, we define 1 < BF_10_ < 3 as anecdotal evidence, 3 < BF_10_ < 10 as moderate evidence, 10 < BF_10_ < 30 as strong evidence, and BF_10_ > 30 as very strong evidence of an effect^26,39^.

Our analysis was conducted in two steps. In the first step, we searched for the best model by comparing models with the null model. In the second step, we analyzed the specific effect of each predictor by calculating the inclusion BFs (BF_incl_) across matched models, representing evidence for all models *with* a term of interest against all models *without* the term. Since we considered matched models only, the models *without* the term included only the main effect terms that constitute the interaction term^40^.

To analyze the proportion of “more negative” responses for a probe set, we used a weakly informative prior, Student’s *t* distribution (ν = 4, µ = 0, s = 2.5), for the regression coefficients of the binary response data. The models were fit using eight chains, each with 6000 iterations, including a warm-up of 1000 samples per chain. This resulted in a total of 40,000 Markov chain Monte Carlo samples. For the RT data, we used a normal prior (*Normal*(0,10)), which is also weakly informative. The models were fit using seven chains, each with 10,000 iterations, including a warm-up of 1,000 samples per chain, resulting in 63,000 Markov chain Monte Carlo samples. The convergence of Markov chain Monte Carlo chains was validated by the Rhat statistic.

Additionally, we conducted a post-hoc analysis on the *either* condition only. Trials of the *either* condition were fit to the Bernoulli distribution with a logit link function. Student’s *t* distribution (ν = 4, µ = 0, s = 2.5) for the regression coefficients was used as a prior, and the models were fit using eight chains, each with 6000 iterations, including a warm-up of 1000 samples per chain. When constructing models, we used two population-level predictors: emotion category (happy, angry) and set with facial masks (probe, control).

We neither excluded nor trimmed specific data or trials when modeling the proportion of “more negative” response data. For the RT data, however, trials with RTs three standard deviations above or below the mean in each condition were considered outliers and subsequently excluded. The exclusion rate was 2% on average across conditions (1.95% in total).

## Results

**Figure 2:**
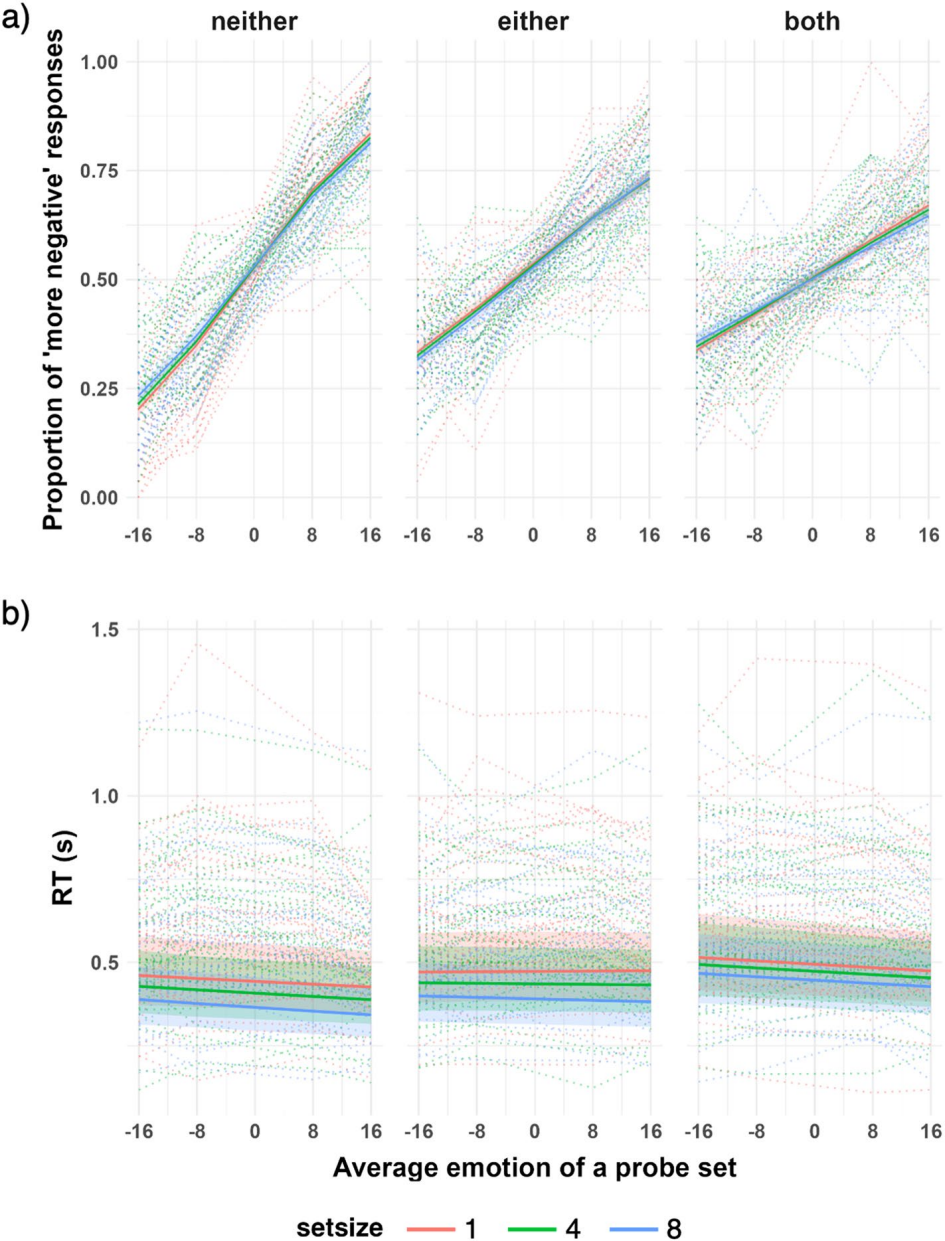
The results of the experiment. *Note*. (**a**) The predicted proportion of “more negative” responses for a probe set, as a function of the average emotion of a probe set (the left end of the x-axis indicates the happiest crowd, and the right end of the x-axis indicates the angriest crowd), using a full Bayesian logistic mixed model. Each graph shows the results of each facial mask condition, depicting how the mask condition affected response patterns. (**b**) The predicted RT using a full Bayesian logistic mixed model. Each graph shows the results of each facial mask condition, indicating how the mask condition affected the RT patterns. In each graph, the pink line indicates the set size 1 condition, the green line indicates the set size 4 condition, and the blue line indicates the set size 8 condition. The shaded areas of each line denote the highest and lowest credible intervals for the estimates. The dotted lines in each graph are the raw data of individuals.

Figure 2 displays the proportion of “more negative” responses (Fig. 2a) for the probe set compared to the neutral control set and the RT (Fig. 2b). Table 1 shows the results of all the Bayesian models tested for finding the best-fitting model. Tables 2 and 3 show the summarized BFs of the effects of interest (i.e., the inclusion BFs).

**Table 1:** Bayesian analysis: model comparison.

	log(BF_10_)	BF_10_
**Models**	**P(Negative)**	**RT**
SetSize	− 6.58	1.33e+33
Emotion	2601.57	140.97
Mask	1.41	1.19e+28
SetSize + Emotion	2595.06	5.98e+35
SetSize + Mask	-5.15	6.22e+61
Emotion + Mask	2604.81	4.94e+29
SetSize + Emotion + SetSize:Emotion	2587.80	1.61e+31
SetSize + Mask + SetSize:Mask	-16.46	4.49e+57
Emotion + Mask + Emotion:Mask	2848.40	2.28e+23
SetSize + Emotion + Mask	2598.31	2.75e+63
SetSize + Emotion + Mask + SetSize:Emotion	2591.04	7.51e+58
SetSize + Emotion + Mask + SetSize:Mask	2587.17	2.09e+59
SetSize + Emotion + Mask + Emotion:Mask	2841.83	1.32e+57
SetSize + Emotion + Mask + SetSize:Emotion + SetSize:Mask	2579.91	5.77e+54
SetSize + Emotion + Mask + SetSize:Emotion + Emotion:Mask	2834.66	3.44e+52
SetSize + Emotion + Mask + SetSize:Mask + Emotion:Mask	2830.78	9.56e+52
SetSize + Emotion + Mask + SetSize:Emotion + SetSize:Mask + Emotion:Mask	2823.50	2.70e+48
SetSize + Emotion + Mask + SetSize:Emotion + SetSize:Mask + Emotion:Mask + SetSize:Emotion:Mask	2810.49	3.36e + 39

All models were compared to the null model, which included only Subject as a random factor. All models also included Subject as a random factor. The log values are reported here because the BFs were too large.

**Table 2:** Analysis of effects (proportion of “more negative” responses).

Effects	P(prior)	P(posterior)	BF_inclusion_
SetSize	0.26	1.40e−03	0.001
Emotion	0.26	1.68e−106	Inf
Mask	0.26	1.61e−106	25.61
SetSize:Emotion	0.26	1.07e−06	7.66e−04
SetSize:Mask	0.26	2.21e−08	1.58e−05
Emotion:Mask	0.26	1	6.19e+105
SetSize:Emotion:Mask	0.05	3.42e−17	2.24e−06

**Table 3:** Analysis of Effects (RTs).

Effects	P(prior)	P(posterior)	BF_inclusion_
SetSize	0.26	1	5.57e+33
Emotion	0.26	0.98	44.29
Mask	0.26	1	4.61e+27
SetSize:Emotion	0.26	2.66e−05	2.72e−05
SetSize:Mask	0.26	7.58e−05	2.58e−05
Emotion:Mask	0.26	4.67e−07	4.77e−07
SetSize:Emotion:Mask	0.05	1.19e−24	1.25e−09

As shown in Table 1, the proportion of “more negative” responses for the probe set was best predicted by the model that contained the main effect terms of emotion and facial mask and the interaction term of the two; this is evidenced by the largest BF (log BF_10_ = 2848.4) when compared to the null model. The inclusion BFs shown in Table 2 provide very strong evidence for the main effect of emotion (BF_incl_ = *Inf*), indicating that the proportion of “more negative” responses increased as the intensity of the angry emotion of the probe set increased.

We also found strong evidence for the main effect of the mask condition (BF_incl_ = 25.61) and very strong evidence for the interaction between the emotion and mask conditions (BF_incl_ = 6.19e+105). The interaction pattern showed that the slopes of the proportion of “more negative” responses (as a function of the intensity of angry emotions in the probe set) varied across the mask conditions (Fig. 2a). The steepest slope was found in the *neither* condition. These results suggest that the perception of a crowd’s average emotion was impaired when faces were wearing masks. Nevertheless, when conducting a simple slope analysis with the mask condition serving as a moderator, the slope estimates at all levels were greater than zero (Table 4). This suggests that masked faces in a crowd weakened the average emotion perception but did not entirely prevent participants from correctly judging the average emotion. Other interaction terms, including a three-way interaction between the set size, emotion, and mask (all BF_incl_ < 0.33), did not provide any significant evidence.

**Table 4:** Simple slopes analysis with mask as a moderator (proportion of “more negative” response data).

Mask condition	Slope estimate	Est. error	CI. lower	CI. upper
Neither	0.09	0	0.086	0.091
Either	0.05	0	0.052	0.057
Both	0.04	0	0.038	0.042

**Figure 3:**
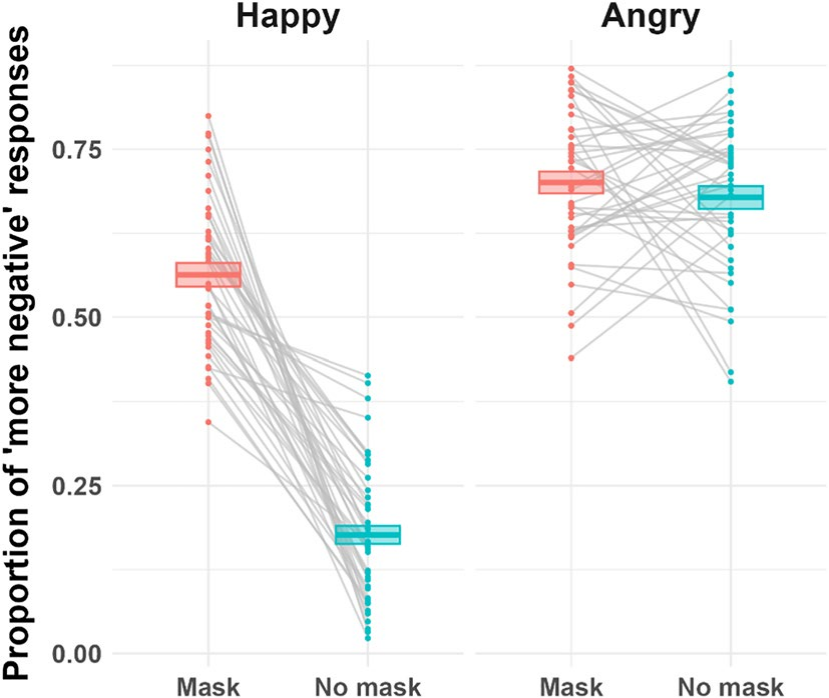
Direct Comparison of the Mask Crowd With the No Mask Crowd (Either Condition). *Note*. In the *either* condition, only one set—either a probe set (i.e., expressive crowd) or a control set (i.e., neutral crowd)—wore facial masks. The x-axis labels indicate the mask condition of the probe set in both the happy and angry emotion conditions. For example, in the left column labeled as “Happy,” the pink box plot indicates the proportion of “more negative” responses assigned to the happy crowd when this crowd was wearing masks and was compared with a control set containing a neutral crowd that was not wearing masks. The blue box plot indicates the proportion of “more negative” responses assigned to the happy crowd when this crowd was not wearing masks and was compared with a control set containing a neutral crowd that was wearing masks.

In the *either* condition, either the probe set or the control set comprised faces wearing masks. We further analyzed the responses in this condition to investigate how participants compared the average emotion of a crowd wearing facial masks with that of a crowd without masks. We collapsed the data across the three levels of set size and the four levels of emotion into two categories: happy and angry. When the average emotion of the probe set was happy, there was a substantial difference in the proportion of “more negative” responses between the case in which the happy crowd wore masks (to be compared with a neutral crowd not wearing masks) and that in which the happy crowd did not wear masks (to be compared with a neutral crowd wearing masks) as shown in the left panel of Fig. 3.

In approximately 53% of the trials, participants judged the happy crowd (probe set) wearing masks as “more negative” than the neutral crowd (control set) not wearing masks. Meanwhile, participants perceived the happy crowd as “more negative” only in approximately 17% of the trials when faces in the happy crowd were not wearing masks and compared with a neutral crowd wearing masks. When the average emotion of the probe set was angry, the proportion of “more negative” responses for the probe set was the same, regardless of whether the crowd in the probe set or control set wore facial masks (right panel of Fig. 3).

The differential effects of facial masks on the perception of happy and angry average emotions suggest that facial masks considerably disrupt the perception of happy expressions to the extent that observers misperceive a happy crowd wearing masks as an angry crowd. These results were confirmed by the inclusion BFs, which indicated very strong evidence for the main effects of the emotion category (BF_incl_ = *Inf*) and the set with facial masks (BF_incl_ = 1.30e+135), as well as the interaction between the two factors (BF_incl_ = 5.43e+103; Table 5).

**Table 5:** Either condition: analysis of effects (proportion of “more negative” responses).

Effects	P(prior)	P(posterior)	BF_inclusion_
EmotionCategory	0.40	1.84e−104	Inf
Mask presence of the emotional crowd	0.40	1.84e−104	1.30e+135
EmotionCategory:Mask presence of the emotional crowd	0.20	1	5.43e+103

The RT data showed very strong evidence for the effect of set size, which is supported by the inclusion BFs (BF_incl_ = 5.57e+33; Table 3). The faster RTs with the larger set sizes (Fig. 2b) suggested that having more faces in a crowd enabled participants to make judgments about average emotions more quickly without affecting the response pattern. The RTs decreased as a function of the average intensity of angry emotion in a crowd, indicating that the more intense the angry emotion was on average, the faster the participants judged a crowd as more negative than a neutral crowd. This observation was supported by very strong evidence for the effect of emotion (BF_incl_ = 44.29). Lastly, there was very strong evidence for the effect of the mask condition (BF_incl_ = 4.61e+27): the RTs increased across the mask conditions in the order of *neither*, *either*, and *both*. The results showed no other supporting evidence for interaction terms (all BF_incl_ < 0.33).

Similar to our analysis of the proportion of “more negative” responses (Fig. 3), we analyzed the RTs in the *either* condition. We found that participants’ RTs were faster for the crowds without masks (BF_incl_ = 2.34e+04; Table 6). However, there was no significant difference between the emotion categories, nor was there interaction between the factors.

**Table 6:** Either condition: analysis of effects (RTs).

Effects	P(prior)	P(posterior)	BF_inclusion_
EmotionCategory	0.40	1.82e−03	0.002
Mask presence of the emotional crowd	0.40	1	2.34e+04
EmotionCategory:Mask presence of the emotional crowd	0.20	5.73e−04	0.296

## General discussion

Reading emotions only from the upper half of faces is more relevant to people’s daily experiences today than ever before. This situation provides important scientific implications on how partial facial information affects the evaluation of different groups of multiple faces in terms of emotional valences and intensities. Everyday items, such as sunglasses, scarves, veils, caps, hats, helmets, and medical masks, lead to the partial obstruction of faces, hampering people’s ability to recognize others’ facial identities and emotions.

However, it has not been tested whether people can still quickly make efficient and reliable judgments about the overall moods of crowds wearing masks, relying only on the visible upper halves of faces. In the present study, we investigated how people’s ability to perceive crowds’ emotions changed when the faces in the crowds varied in terms of emotional valence, intensity, and crowd size. We found that wearing masks impaired people’s ability to judge the average emotions of crowds, especially happy crowds: Happy crowds that wore masks were likely to be judged as negative. Facial masks reduced the sensitivity and speed with which participants perceived the overall mood of a facial crowd. Nevertheless, people could correctly distinguish the emotional intensities of facial crowds wearing masks better than chance. As the crowd size increased, participants could judge the crowd’s overall mood faster with similar accuracy.

When the bottom halves of people’s faces are hidden by facial masks, one’s overall mood judgments of facial crowds can be systematically biased to be more negative than when people do not wear masks. Social communication could be improved if individuals who wear masks were to be over-expressive and convey their emotions to compensate for wearing a mask. However, facial features contribute to emotional perceptions in different ways depending on which facial expression is expressed. Beaudry et al.^41^ showed that hiding the mouth area disrupted the categorization of happy expressions more than hiding the eye/brow area; the opposite trend was observed for angry expressions. Furthermore, hiding the eye/brow region did not affect happy emotion categorization, whereas it disrupted angry emotion categorization. Although East Asians tend to distribute their fixations around eye areas to judge facial expressions more than Westerners^42^, the effect of hiding the mouth or eye/brow areas on categorizing happy and angry faces was similar between Canadian^41^ and Korean observers^43^. Our results are consistent with these previous findings, as we found that hiding the mouth region with a mask impaired the discrimination of the intensity of the angry and (especially) the happy emotions of a crowd.

Our results suggest that concealing the mouth area hampers not only the emotion categorization of a single face but also the ensemble emotion judgment of multiple faces. These findings suggest that the quality of individual members’ representations significantly impacts ensemble perception. Previous research also showed that reduced individual emotion intensities due to face inversion impaired observers’ overall emotion intensity judgments^24^. Conversely, when the fast flicker adaptation enhanced individual emotion perception, the sensitivity of overall mood judgments increased, both for happy and angry expressions^44^. Additional evidence supports the finding that there is a strong relationship between ensemble face processing and individual face processing. When specific facial information (such as gaze direction) cannot be ignored during the categorization of individual face emotion^45^, the same applies to ensemble face processing, as evident in the significant error correlation between ensemble representations of gaze directions and facial expressions^46^. The facial information extracted from a single face transfers to the processing of an ensemble of faces. These findings underscore the interdependence of individual face processing and ensemble perception, highlighting how the properties of individual faces shape the overall perception of the emotion of a crowd of those individuals.

Ensemble representations are a form of efficient coding that allows observers to create a rapid, unified impression of multiple individual elements. This ensemble coding shows the wisdom of crowds effect, indicating that when measurements are averaged, benefits arise because the errors caused by noisy representations of individuals cancel out each other^24,47–54^. Thus, the benefits of averaging likely depend on the number of individual measurements averaged, with more measurements resulting in greater benefits. Our results confirm the power of averaging by showing that the more individual faces are present in a crowd, the more quickly participants can respond without sacrificing accuracy. This is the case even when perceiving the emotions of individual faces becomes more challenging when people are wearing facial masks. In addition, that participants’ performance is unaffected by the increasing set sizes suggests that ensemble coding can be achieved in parallel. The facial expressions extracted from a group of people are useful for promoting communication with others and regulating subsequent social behaviors^16,18^. A person may react more quickly to a social context involving a large than a small group, but further research is required to empirically test this. Altogether, the current study provides a novel implication: under ambiguous social situations where masks interfere with the perception of others’ emotions, perceiving a set of faces could be more advantageous than relying on an individual face.

Previous studies have found set size effects in the opposite direction from our results, particularly in crowds of faces^55,56^. Ji and Pourtois^55^ found that the accuracy of averaging facial expressions decreased as the set size increased. The side-by-side presentation might have decreased the accuracy of a larger set due to confounding factors such as crowding and cluttered display. In contrast, in the current study, we presented the two facial crowds sequentially so that individual faces were less likely to be crowded or cluttered. Another possibility is the interaction between set size effects and stimuli variability. The visual system cannot process more information than its capacity limit. The capacity limit constrains the effect of noise cancellation in large sets, which is reflected in the late noise of averaging^48,54^. When stimuli variability is high, the effect of noise cancellation as the set size increases is weakened because the stimuli are not densely centered around the representative value. Even in the work of Ji and Pourtois^55^, when emotion variance was low, the ability to average facial expressions did not depend on set size.

In the present work, we investigated how people’s overall judgments of the mood of a crowd are influenced by the occlusion of part of the faces with masks. We found that participants’ judgments were impaired by masks, and such impairment was more pronounced for happy emotions than for angry emotions. Nevertheless, due to the power of averaging, observers could determine the overall mood of a large crowd more quickly than a small crowd. Altogether, our results suggest that people’s ability to apprehend social-emotional contexts and react to them can be efficiently aided by large crowds, even when individual emotional representations are impaired or partially occluded.

### Supplementary Information

Supplementary Table 1.

## Data Availability

The datasets generated and/or analyzed during the current study are available in the Open Science Framework repository, https://osf.io/75zwr.
