# Supplementary material for: The effect of masks on the emotion perception of a facial crowd
Source: Sci Rep. 2023 Aug 31;13:14274. doi: 10.1038/s41598-023-41366-0 (PMC10471755; doi:10.1038/s41598-023-41366-0)
Supplement: Supplementary file 1 — Supplementary Table 1. [file 41598_2023_41366_MOESM1_ESM.docx]

The Effect of Masks on the Emotion Perception of a Facial Crowd

Jieun Cho, Hee Yeon Im, Young Jun Yoon, Sung Jun Joo, Sang Chul Chong

Supplementary Table 1

*Intensity ratings and emotional categories for face stimuli used in the present study.*
